# Application of a Novel Functional Gene Microarray to Probe the Functional Ecology of Ammonia Oxidation in Nitrifying Activated Sludge

**DOI:** 10.1371/journal.pone.0077139

**Published:** 2013-10-14

**Authors:** Michael D. Short, Guy C. J. Abell, Levente Bodrossy, Ben van den Akker

**Affiliations:** 1 UNSW Water Research Centre, School of Civil and Environmental Engineering, the University of New South Wales, Kensington, New South Wales, Australia; 2 SA Water Centre for Water Management and Reuse, School of Natural and Built Environments, University of South Australia, Adelaide, South Australia, Australia; 3 CSIRO Marine and Atmospheric Research and Wealth from Oceans National Research Flagship, Hobart, Tasmania, Australia; Catalan Institute for Water Research (ICRA), Spain

## Abstract

We report on the first study trialling a newly-developed, functional gene microarray (FGA) for characterising bacterial and archaeal ammonia oxidisers in activated sludge. Mixed liquor (ML) and media biofilm samples from a full-scale integrated fixed-film activated sludge (IFAS) plant were analysed with the FGA to profile the diversity and relative abundance of ammonia-oxidising archaea and bacteria (AOA and AOB respectively). FGA analyses of AOA and AOB communities revealed ubiquitous distribution of AOA across all samples – an important finding for these newly-discovered and poorly characterised organisms. Results also revealed striking differences in the functional ecology of attached versus suspended communities within the IFAS reactor. Quantitative assessment of AOB and AOA functional gene abundance revealed a dominance of AOB in the ML and approximately equal distribution of AOA and AOB in the media-attached biofilm. Subsequent correlations of functional gene abundance data with key water quality parameters suggested an important functional role for media-attached AOB in particular for IFAS reactor nitrification performance and indicate possible functional redundancy in some IFAS ammonia oxidiser communities. Results from this investigation demonstrate the capacity of the FGA to resolve subtle ecological shifts in key microbial communities in nitrifying activated sludge and indicate its value as a tool for better understanding the linkages between the ecology and performance of these engineered systems.

## Introduction

Biological wastewater treatment systems are in essence engineered extensions of natural eutrophic ecosystems. Activated sludge (AS) processes in particular are highly engineered, forced ecosystems that rely almost exclusively on complex microbial communities to catalyse the dominant steps of nitrogen removal. Until quite recently, developments in these wastewater treatment processes have occurred largely without regard to—or a thorough understanding of—the key organisms involved. It is widely acknowledged that further optimisation of AS process stability and performance requires an improved understanding of the relationships between the fundamental microbiology and process engineering aspects of these systems [1-4]. As such, scientists and engineers should share a common interest in better understanding the functional ecology of wastewater treatment systems in order to better exploit these linkages to satisfy future process design and optimisation objectives [1]. 

Meaningful insights into the functional ecology of AS systems cannot be obtained by traditional ‘bulk parameter’ measurements alone; instead they require targeted, high-resolution molecular microbiological methods [4]. Furthermore, traditional culture-based assays such as most probable number and selective cultivation methods for detecting nitrifying microbes in environmental samples are both time-consuming (due to the slow growth rates of these organisms) and erroneous (due to sub-optimal culture conditions), resulting in a misleading representation of the target microbial community. Since the performance of AS processes are largely determined by the nature and activity of resident microbes, developments in microbiological methods and understanding have historically been the limiting factor in advancing this technology. In recent times, molecular biology has revolutionised the way in which key microbial populations are able to be assessed in engineered biological systems and have greatly advanced our understanding of the links between the fundamental microbiology and functional performance of these systems [1,4]. With this in mind, tremendous scope exists for the development of molecular-based monitoring tools for optimising the functional performance of wastewater treatment systems.

The complicated configuration of engineered BNR systems makes controlling process performance difficult and for plants with combined nitrification–denitrification, failure to maintain stable nitrification performance is a well-known problem that stems from the finicky nature of the microbes involved [3,5]. In nitrifying AS, ammonia oxidation is the rate-limiting process and ammonia monooxygenase is the key enzyme involved. The molecular ecology of ammonia oxidation has been most extensively explored by surveying the functional gene (*amoA*) encoding for the α-subunit of ammonia monooxygenase [6,7]—the enzyme responsible for catalysing the first step of ammonia oxidation during primary nitrification. One tool capable of such exploratory analyses is the functional gene microarray (FGA). Microarray technology allows for highly parallel detection and semi-quantitative characterisation of target genes from multiple organisms at a high taxonomic resolution, thereby facilitating the characterisation of complex microbial communities in a wide range of environmental matrices [4,8]. Microarrays offer unique advantages over conventional methods, such as DGGE and T-RFLP. For example, they allow assignment of putative identification to the detected organisms, enable high-throughput analysis not possible with methods such as FISH and conventional clone libraries, and also represent a more simple, lower cost approach than modern next-generation sequencing methodology [8]. In the context of wastewater research, FGAs offer the potential for economical, high-throughput, high-resolution screening of large numbers of samples from different treatment configurations. In return they provide detailed information on the nature of key microbial communities, such as those involved in nitrogen removal, and facilitate the exploration of links between these communities and more conventional indices of treatment performance. 

Since DNA microarrays were first used some two decades ago to profile gene expression in *Arabidopsis* [9], only a handful of studies have since applied microarray-based methods to characterise nitrifying communities in AS [10-12] and none have encompassed archaeal nitrifiers. Accordingly, the aim of this study was to apply a novel, state-of-the-art FGA [13] to characterise both bacterial and archaeal ammonia oxidisers in a full-scale integrated fixed-film media activated sludge (IFAS) plant. The FGA applied here is currently the only operational microarray-based tool comprehensively targeting the entire known diversity of aerobic ammonia-oxidising microbes (both cultivated and un-cultivated). 

## Methods

### Plant sampling and description

Mixed liquor (ML) grab samples and attached biofilm (media) samples were collected on seven occasions from the aeration zone of a full-scale, nitrifying IFAS plant located in metropolitan Adelaide, South Australia between August and November of 2011 (Austral winter–spring). During each sampling event, one litre grab samples of ML and four plastic media carriers were collected and immediately frozen (−80°C) until further processing. A comprehensive description of the study IFAS plant can be found elsewhere [14]. Briefly, the plant was operated in a pre-denitrification–nitrification configuration, with effluent recycling (100–300%) to the front of the plant. Solids retention time (SRT) was 6.7 days, mixed liquor suspended solids (MLSS) was maintained at 2283 mg L^−1^ and the sludge loading rate (SLR) was 0.14 kg BOD_5_ kg MLSS^−1^ d^−1^. Dissolved oxygen levels in the aerobic zones were tightly controlled at 5 mg L^−1^ in the first aerobic zone, followed by 2 mg L^−1^ in the subsequent zone. Molasses was dosed for enhanced denitrification. Summary statistics of IFAS plant operating conditions and performance are given in Table 1. All necessary permits were obtained for the field study described here. Permission to enter the wastewater treatment facility and for the collection of activated sludge samples from the IFAS system was granted by the managing water authority (SA Water Corporation).

**Table 1:** Summary statistics for the operational and performance data of the study IFAS plant during the four month monitoring period.

	IFAS influent			IFAS mixed liquor				IFAS effluent		
	NH_4_^+^-N	Temp.	TDS	SLR	MLSS	SRT	SVI	NH_4_^+^-N	NO_3_^−^-N	TN
	(mg L^−1^)	(°C)	(mg L^−1^)	(kg BOD_5_ kg MLSS^−1^ d^−1^)	(mg L^−1^)	(d)	(ml g^−1^)	(mg L^−1^)	(mg L^−1^)	(mg L^−1^)
Mean	42	21	1225	0.14	2283	6.67	342	0.60	7.16	11
Min.	37	20	1200	0.12	2190	6.50	303	0.13	5.93	10
Max.	47	22	1300	0.17	2410	6.94	388	2.95	8.20	11

### Functional gene microarray (FGA)

**Figure 1:**
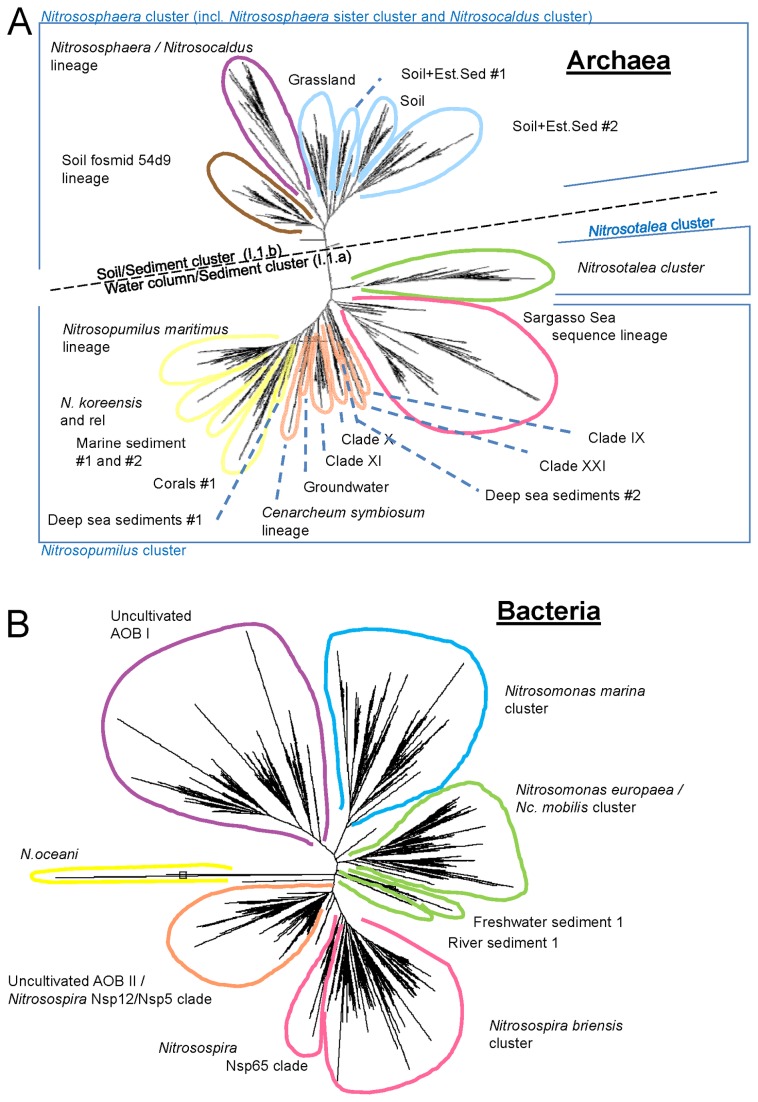
Radial phylogenetic trees of: (A) archaeal; and (B) bacterial *amoA* sequences targeted by the FGA (after Abell et al. [13]).

A state-of-the-art, high-throughput FGA [13] was employed to assess the relative abundance and diversity of both ammonia-oxidising bacteria (AOB) and archaea (AOA) in the nitrifying IFAS samples. Biomass isolated from each sample was used for DNA extraction according to a previously developed method [15] and our methodology here followed that described elsewhere for environmental sample analyses [13]. The microarray consists of a small solid substrate (glass microscope slide) to which a set of targeted oligonucleotide probes is attached. The functional gene of interest (*amoA*) is amplified from DNA extracted from the wastewater samples, fluorescently labelled and is then applied to the FGA under specific conditions to produce fluorescent probe–target duplexes which can be detected and analysed. The FGA [13] targets the functional *amoA* gene encoding subunit A of the ammonia monooxygenase enzyme and is based on a previously developed and proven method for methane-oxidisers [16]. The *amoA* gene in particular is widely recognised as both an effective phylogenetic marker and a specific functional marker for detecting ammonia-oxidising microbes in the environment [4] and has been most extensively studied relative to its enzyme subunit B and C-encoding counterparts (*amoB* and *amoC*) [7]. The FGA targets the entire known diversity of autotrophic AOB and AOA (both cultivated and un-cultivated); the phylogenetic coverage of the FGA is shown in Figure 1. All raw data is available from the authors upon request.

### Real-time polymerase chain reaction (qPCR)

In parallel to microarray analyses, the abundance of bacterial and archaeal *amoA* genes was also assessed for all samples using qPCR according to previously described protocols [17]. Briefly, the abundance of AOB or AOA *amoA* was evaluated in separate assays in triplicate for each sample by comparing to a dilution series of gene copies of known abundance. Measures were taken to ensure there were no confounding effects of PCR inhibitors in the samples and reactions were inspected using agarose gel electrophoresis after each assay.

### Statistical analyses

Multivariate statistical analyses of the microarray data was performed using PRIMER 6 (Primer-E, Plymouth, UK). Universal and control probe signals were firstly removed from the analysis and then probe signals were standardised by sample and later square root transformed. Diversity of each group (AOB or AOA) was assessed by comparing the Shannon diversity indices (*H*’) of microarray signal outputs for each sample, with higher values corresponding to a higher diversity of the target group in the sample. Non-metric multidimensional scaling (MDS) (1000 permutations) was used to represent community differences between samples. Analysis of similarity (ANOSIM) was also used to test for differences in the structure of AOB and AOA communities between the IFAS media and ML fractions. AOB and AOA community structure was also compared with operational parameters by way of non-parametric BIO-ENV analysis [18] in order to identify operational parameters that were significantly associated with sample similarities of the microbial community. Bivariate correlation analyses were performed in the R package [19] to distil relationships between ammonia oxidiser functional gene abundance and (rolling seven day average) IFAS plant performance. 

## Results and Discussion

### IFAS plant ammonia oxidiser ecology

**Figure 2:**
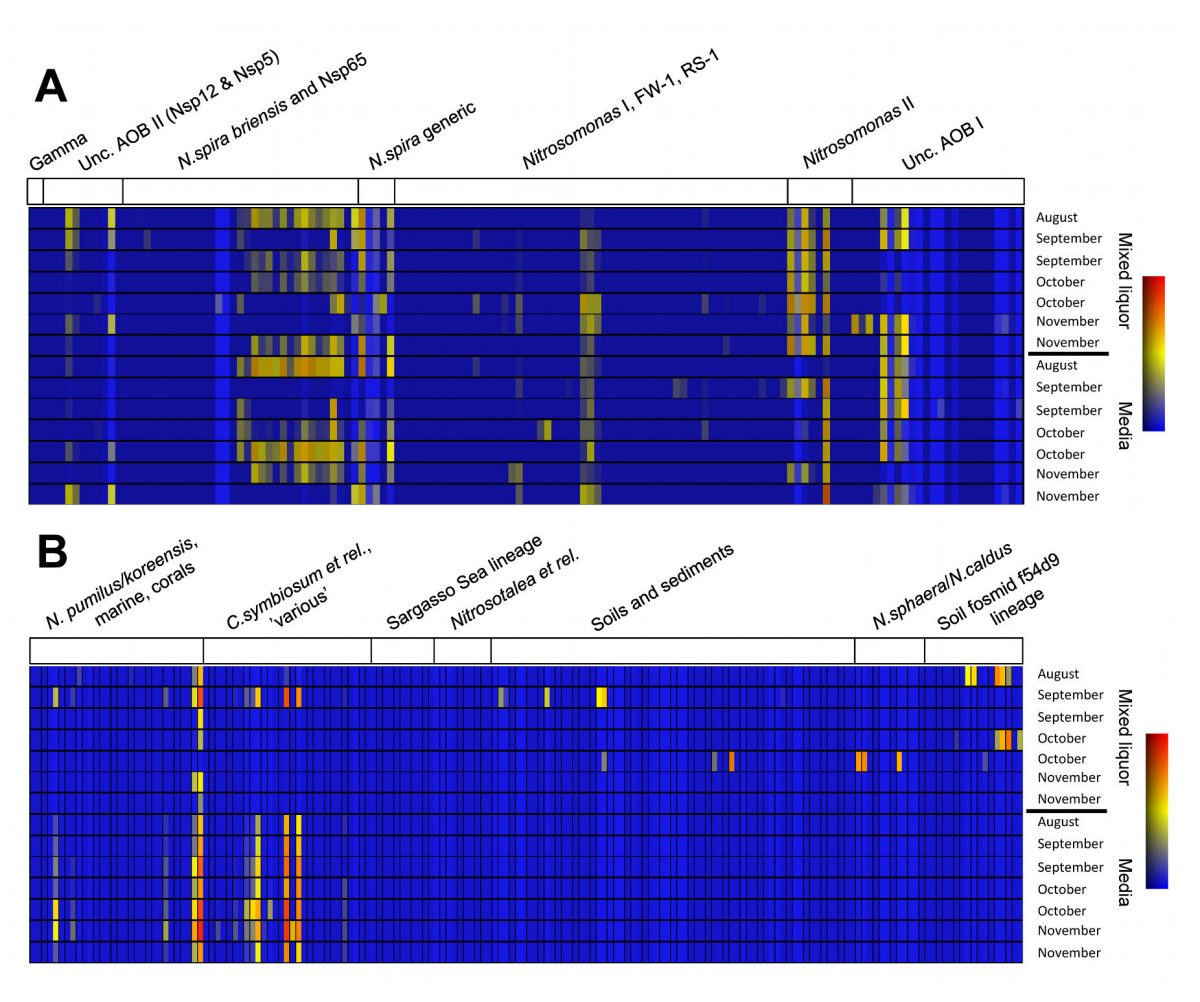
FGA results showing temporal changes in ammonia oxidisers in the mixed liquor (top) and media (bottom) fractions of the surveyed IFAS plant: (A) AOB; (B) AOA. Colour gradient (blue–yellow–red) depicts relative abundance on log scale (0–0.1–1.0 respectively).

Results from this full-scale investigation represent the first demonstration of a newly-developed FGA for characterising the diversity of both AOB and AOA in activated sludge. Figure 2 illustrates how the results from the FGA analysis are outputted graphically. These figures allow for immediate, semi-quantitative comparison of the relative abundance and diversity of key organisms and as such, provide an intuitive and comprehensive first assessment of temporal shifts in ammonia-oxidising communities. 

**Figure 3:**
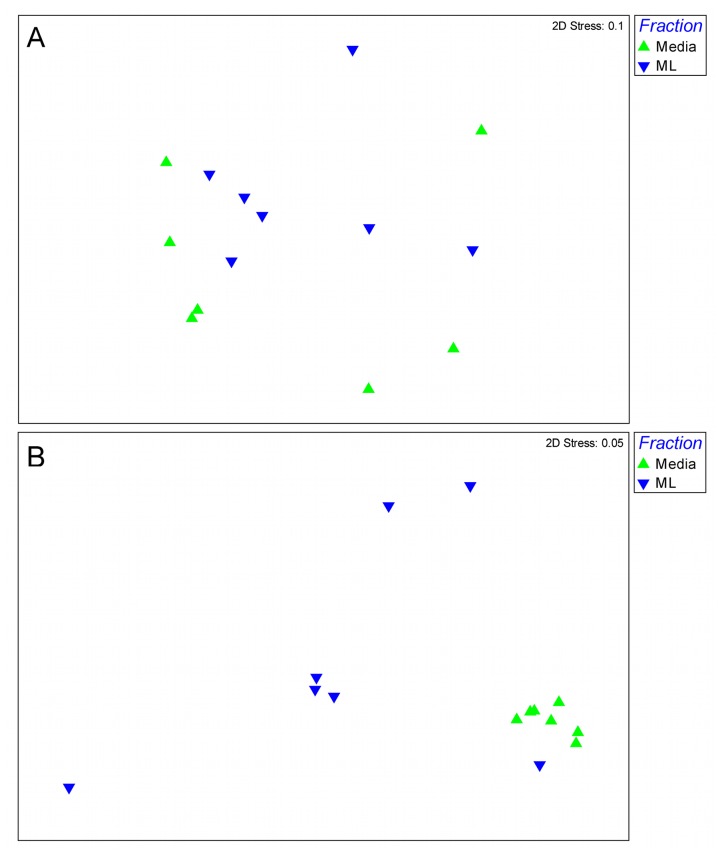
MDS analysis of square root-transformed microarray data, comparing AOB (A) and AOA (B) in complementary mixed liquor (ML) and IFAS media (Media) fractions. Stress values for ordination are shown in the upper right corner of each plot.

As anticipated, the AOB community across all samples was strongly represented by species of *Nitrosospira* and *Nitrosomonas* (Figure 2A); these organisms are widely considered to be the two most important genera of AOB in many biological nitrogen removal systems including AS [4]. Aside from this more conventional insight, the AOB community was also found to be strongly represented by members of uncultivated clades (Figure 2A). This observation has important practical implications for past and also future investigations into the ecology of nitrifiers in wastewater environments, since uncultivated organisms may be unaccounted for by conventional molecular approaches (e.g., FISH and other 16S-based methods) due to an unknown and likely highly divergent 16S rRNA gene affiliation that makes it next to impossible to identify the physiology of these organisms. Failure to recognise and account for this in future such research may result in a distorted picture of the resident communities of interest. Figure 2A also shows that despite being confined to several dominant genera, the IFAS reactor AOB community was relatively diverse in terms of the number of phylotypes represented (mean *H’* of 3.3 and 3.1 for ML and media AOB respectively). Others have suggested that particularly high and low NH_4_^+^-N concentrations generally constrain AOB diversity in wastewater systems [5] such that the relatively high AOB diversity here may be a consequence of the moderate NH_4_^+^-N levels in our domestic wastewater (Table 1). In terms of the distribution of AOB between the two sub-compartments of the IFAS reactor (i.e. ML and media), the FGA output identified similar community ecologies; this can be seen qualitatively in the apparent ‘mirroring’ of the upper and lower panes of Figure 2A and is further reflected in the lack of separation of the two reactor compartments during multivariate ordination (R = 0.07, *p* > 0.1; Figure 3A) as well as the similar community diversities (1-way ANOVA; *p* = 0.66). 

The AOA community in the ML phase was dominated by organisms related to *Nitrosopumilus maritimus*; these organisms were also highly abundant in the IFAS media (Figure 2B). AOA grouping with *Cenarchaeum symbiosum*, whilst abundant in the IFAS media, were rarely detected in the mixed liquor (Figure 2B). AOA found exclusively in the mixed liquor included groups related to *Nitrososphaera gargensis* and other organisms closely related to Archaea previously isolated from soil and sediment environments. Results here for AOA community composition broadly reflect those of prior wastewater treatment investigations [7,20-22]. Contrasting Figure 2B with Figure 2A, diversity in the AOA community was observed to be somewhat constrained relative to that of AOB (Table 2); although this trend was significant only for the ML fraction (1-way ANOVA; *p* = 0.03). These results echo the findings of wastewater research internationally [21-23] wherein the diversity of AOA *amoA* sequences recovered from a given treatment system has generally been low. This finding may also serve to complement broader environmental observations of a tendency for strong habitat-partitioning of archaeal *amoA* diversity [20,23] and the emerging consensus of preferential niche specialisation in AOA [6,24,25]. 

**Table 2:** Results from temporal qPCR analyses of bacterial (AOB) and archaeal (AOA) *amoA* copy numbers in the respective IFAS plant fractions (mixed liquor (ML; copies ml^−1^) and media (Media; copies cm^−2^ of surface area) given alongside relevant plant performance data.

Sample month	AOB - ML			AOA - ML			AOB - Media			AOA - Media			NH_4_^+^-N^a^	NO_3_^−^-N^a^	% NH_4_^+^-N removal
	*amoA*/ml	*amoA*/ng DNA	Diversity (*H*’)	*amoA*/ml	*amoA*/ng DNA	Diversity (*H*’)	*amoA*/cm2	*amoA*/ng DNA	Diversity (*H*’)	*amoA*/cm2	*amoA*/ng DNA	Diversity (*H*’)			
Aug.	2.9×10^6^	8.0×10^2^	3.42	1.6×10^5^	4.3×10^1^	3.15	1.4×10^5^	3.0×10^2^	3.41	2.5×10^5^	5.3×10^2^	3.22	0.13	7.50	99.7
Sept.	5.5×10^6^	1.2×10^3^	3.26	2.9×10^5^	6.5×10^1^	3.43	2.0×10^5^	2.6×10^2^	2.83	1.1×10^4^	1.5×10^1^	3.20	0.43	8.18	99.1
Sept.	4.7×10^6^	1.0×10^3^	3.17	2.2×10^5^	4.8×10^1^	2.62	6.5×10^4^	1.6×10^2^	2.87	1.0×10^5^	2.6×10^2^	3.28	2.95	5.93	92.1
Oct.	4.4×10^6^	1.9×10^3^	3.30	1.7×10^5^	7.5×10^1^	3.08	8.4×10^4^	1.7×10^2^	3.23	6.4×10^4^	1.3×10^2^	3.25	1.83	6.13	95.7
Oct.	3.7×10^6^	1.4×10^3^	3.19	2.0×10^5^	7.6×10^1^	2.76	1.1×10^5^	2.3×10^2^	3.34	3.0×10^5^	6.3×10^2^	3.59	0.85	6.93	97.8
Nov.	1.2×10^6^	7.3×10^2^	3.21	7.4×10^4^	4.8×10^1^	2.53	1.3×10^5^	3.5×10^2^	3.14	1.1×10^5^	3.0×10^2^	3.66	1.03	7.15	97.4
Nov.	1.3×10^6^	5.4×10^2^	3.31	1.2×10^5^	5.0×10^1^	2.88	1.1×10^5^	3.1×10^2^	3.02	8.1×10^4^	2.3×10^2^	3.16	0.24	7.25	99.4

a Dissolved nitrogen concentration in IFAS plant final effluent (mg L^−1^).

Despite the rapidly expanding body of international research into the Thaumarchaeota, we are only just beginning to probe the general ecophysiology, metabolic and biogeochemical functionality of these organisms. The contribution of AOA to nitrogen removal during wastewater treatment operations in particular remains largely unexplored and is poorly understood. Results from our analysis demonstrate the persistence of AOA in all collected samples and echo recent international findings [22,26,27], suggesting that these organisms are not only widespread but are likely contributors to the processing activity of some urban wastewater treatment operations. At the same time, geographically-integrated surveys of wastewater treatment operations have demonstrated a patchy distribution of (putative) archaeal ammonia oxidisers between study sites [21,28], indicating that AOA are more important for nitrogen removal at some treatment plants than others. At the same time, the metabolic fundamentals of so-called ‘*amoA*-encoding’ AOA remain unclear. For example, Mußmann et al. [28] have suggested that some AOA are not true autotrophic ammonia oxidisers, while other research indicates a capacity for mixotrophic growth [29] or confirms obligate chemolithoautotrophy among AOA [30]. From a functional ecology perspective, results from the FGA analysis here indicate that an unknown portion of the ammonia-oxidation load in this IFAS plant is likely to be undertaken by ammonia oxidisers that are currently ignored or at best vastly under-represented by many commonly applied analytical methods. Moreover, an unknown fraction of ammonia oxidation in this system is being performed by species of AOB and AOA about which we know very little. These finding have significant practical implications for any future studies aimed at better understanding the functional ecology of wastewater systems in general and highlights the need to apply broad-spectrum ecological tools such as the FGA here in order to capture the full diversity of key communities of interest. 

### Temporal and spatial ammonia oxidiser dynamics

Figures 2A and B demonstrate how the FGA allows for the rapid detection of temporal shifts in the composition of target microbial communities. For example, it can be quickly established that temporal changes in AOA communities during the four month monitoring program were most pronounced in the ML phase whereas the attached biofilm community of the IFAS media was comparatively stable (Figure 2B). This qualitative observation was supported by a significant divergence in AOA community diversity between the two reactor compartments (1-way ANOVA; *p* < 0.01). While the exact origins of this observation remain unclear, we suspect it is a product of the physical configuration of the IFAS reactor and also an assumed preference for media-attached growth in AOA relative to suspended ML growth—aspects discussed in more detail below. 

In contrast to the trends described for AOA above, more notable temporal shifts in the AOB community were observed not only in the ML but also in the media fraction (Figure 2A). The most apparent shifts were seen for AOB of the genera *Nitrosospira* and *Nitrosomonas* in both the ML and media fractions of the IFAS reactor. Various uncultivated AOB were also well represented in both the ML and media communities and were present across many of the sampling intervals, especially within the ML phase. How much of these observed dynamic shifts in AOA and AOB communities resulted from allochthonous microbial influx of sewage-borne populations and how much is driven *in situ* through variations in reactor operating conditions (at both daily and sub-daily timescales) remains unknown; the raw influent wastewater was not monitored for ammonia oxidisers. The factors shaping AOA and AOB community succession in AS systems of all configurations and their relationship to plant performance parameters should be the subject of further investigation. 

Results from multivariate analyses contrasting AOB and AOA communities within the suspended ML and attached media fractions of the IFAS plant are given in Figures 3A and B respectively. As indicated during earlier discussions of the graphical FGA outputs (Figure 2A), results of this analysis confirmed that bacterial ammonia oxidiser communities of both IFAS reactor fractions were indeed similar (R = 0.065, *p* > 0.1; Figure 3A), suggesting a relatively homogeneous distribution of resident AOB throughout the entire IFAS reactor. Conversely, results from the parallel AOA analysis showed that the ML and media biofilm fractions were significantly different in their archaeal ecologies (R = 0.55, *p* < 0.01; Figure 3B). This analysis provided further quantitative support for the assertions made earlier regarding the contrasting nature of ML and media-attached AOA communities as revealed by the FGA (Figure 2B) and again suggest a strong divergence in AOA community ecology between the two reactor compartments. 

It is difficult to put the above observations in a broader literature context, since we are unaware of any comparably comprehensive investigations of ammonia oxidiser ecology in IFAS systems; we reiterate that prior studies of nitrifiers in IFAS systems have neglected AOA [3,31-35]. It is tempting to speculate, however, that the environmental conditions likely to persist within the IFAS media biofilm (i.e. strong vertical gradient of substrate O_2_ and NH_4_^+^-N and possibly elevated C/N ratio) may drive niche differentiation of AOA over AOB. This hypothesis is in part supported the growing evidence base indicating that AOA are more competitive under low NH_4_^+^-N and/or low O_2_ conditions in wastewater treatment situations [21,22,27,36,37] as well as in other aquatic environments [38]. These observations are further supported by the recognised substrate mass transfer limitations in IFAS media biofilms [33,39] and the comparatively lower half-saturation constants of AOA for substrate NH_4_^+^-N and O_2_ [25,37,40]. Another possible explanation for the observed preference of AOA for media-attached growth relates to the physical configuration and operation of IFAS plants, wherein the presence of attached-growth biomass yields an inherently longer sludge age (SRT) for these systems relative to the ML phase [34], potentially providing enhanced opportunities for slower growing/less-competitive AOA to establish. Elsewhere, SRT has been shown to correlate positively with archaeal *amoA* recovery from activated sludge systems [21]. The overall effect of these factors is either an environmental niche preference of AOB for suspended growth and/or competitive displacement of AOB by AOA within the media-attached biofilm. In the context of our study system, the stark physicochemical differences between the suspended ML phase and IFAS media environments (e.g., substrate O_2_ and NH_4_^+^-N availability alongside obvious physical differences) are most likely having a determining influence on the ammonia oxidiser ecology of the two reactor compartments. To what extent the augmented environmental niche diversity in IFAS systems relates to ecophysiological niche diversity in ammonia oxidiser communities, and what effect this then has on reactor operation, performance and stability remains unclear; others report improved nitrification capacity and stability in IFAS relative to parallel conventional AS [34]. An improved understanding of these factors should ultimately have real implications for the operation and performance of these treatment systems. 

### Functional community diversity versus IFAS plant performance

Exploratory multivariate analysis of the composition of AOB and AOA communities showed no obvious relationships between community composition and the operational plant performance variables of Table 1 (BIO-ENV, *p* > 0.05). Contrasting Figure 2A and 2B with the process performance data of Table 1 and 2 reveals some considerable temporal shifts in AOB and AOA community composition against a backdrop of consistently high nitrification performance (mean NH_4_^+^-N removal >97%). These findings imply either some degree of functional redundancy in AOB and/or AOA communities in the study IFAS reactor (i.e. surplus nitrification capacity), or may be an artefact of dataset size and/or monitoring duration. Recent work by Valentín-Vargas et al. [41], for example, suggests an important role for functional redundancy in microbial communities in the maintenance of functional stability during AS treatment. Furthermore, key operational parameters here were very tightly controlled or exhibited little natural variation (Table 1) such that reactor conditions may not have been sufficiently dynamic to reveal measurable links between community composition and process performance. 

Following a long-term whole-of-community survey of AS bacterial ecology at the full-scale, Wells et al. [42] suggest that microbial communities may simply exhibit a baseline of moderate population dynamics not necessarily reflected in plant performance, with periodic dramatic shifts in community structure possibly impacting on the functional stability and process performance of these systems. While operational and environmental factors do have a determining influence on the ecology of AS communities, random immigration and chance effects may also play a dominant role in shaping ammonia oxidiser communities in these systems [43]. Earlier, Briones and Raskin [1] presented evidence to suggest that the levels of individual microbial populations fluctuate irrespective of the functional stability of a bioreactor community. More recent work, however, has found evidence of deterministic factors shaping microbial communities in full-scale AS reactors [41]. Despite this research, the extent to which diversity in functional microbial communities influences the functional stability and/or performance of engineered bioreactors remains largely unresolved [1,4,41,42,44]. To further distil these factors, studies are needed contrasting functional microbial diversity with plant performance parameters during non-steady-state operating conditions (e.g., commissioning phase or during significant process oscillations and/or failures), ideally at full-scale. Extended monitoring programs are also required to better encapsulate possible seasonal dimensions in the above. 

### Functional gene abundance versus IFAS plant performance

Since the FGA provides information on the proportional abundance of different ammonia oxidisers rather than absolute abundance, parallel qPCR of bacterial and archaeal *amoA* genes in both reactor fractions was performed to further distil possible relationships between ammonia oxidiser abundance and plant performance. These results are presented in Table 2. AOB and AOA functional gene abundance in both the ML and IFAS media was quite stable throughout the four month monitoring period, fluctuating by less than one order of magnitude during this time. Observed mixed liquor *amoA* gene abundances for AOA and AOB here (^≈^10^5^–10^6^ ml^−1^) (Table 2) were also within the range of those reported elsewhere for activated sludge bioreactors (^≈^10^4^–10^8^ ml^−1^) [27,37]. Since the primary ML and media gene abundance data are presented in different units (i.e. volumetric versus areal), data are also presented per unit DNA. A comparison of the bacterial-to-archaeal *amoA* gene ratios in the ML with those of the IFAS media biofilm (Table 2) reveals a divergence in relative ammonia oxidiser functional gene abundance between these reactor compartments. For the ML phase, the mean ratio of AOB *amoA*/AOA *amoA* was ^≈^19:1 whereas for the media biofilm, the same ratio was one order of magnitude lower at just 3:1. These results indicate on the whole that AOB functional genes predominate throughout the IFAS reactor and that this dominance is markedly more pronounced in the ML phase than in the media. Interestingly, *amoA* abundance for AOB and AOA was positively correlated in the ML phase of the plant (*r* = 0.93; *p* = 0.003) but no such relationship was observed for the media fraction (*p* > 0.7). 

Contrasting these results with those described earlier (Figure 2B) it appears that AOA inhabiting the IFAS media biofilm may be functionally different to those in the ML. Earlier analysis of the FGA data (Figure 3B) revealed that ML and media biofilm fractions were significantly different in their archaeal ecologies; this observation is supported in principle by qPCR data here in terms of the strong divergence in AOB/AOA *amoA* ratios between the two reactor compartments (Table 2) combined with the absence of AOB–AOA *amoA* covariance for IFAS media data only. Earlier we described how exploratory multivariate analyses revealed no obvious (*p* < 0.05) relationships between ammonia oxidiser community composition and operational plant data. Subsequent correlation analyses, however, revealed some apparent relationships between relevant indices of plant nitrification performance (% NH_4_^+^-N removal and effluent NO_3_^−^-N concentration) and media biofilm AOB *amoA* abundance (Table 2) (*p* = 0.03 and *p* < 0.001 respectively). 

Comparable research in this area generally reports a trend of greater AOB abundance and/or nitrification activity in IFAS media relative to the ML fraction [33,34,45]; although this trend is apparently not universal (e.g., Li et al. [32]). The above correlations, in particular the positive association between media biofilm AOB *amoA* abundance and %NH_4_^+^-N removal, complement the general findings of prior IFAS studies. In the context of our IFAS system, however, these relationships seem somewhat counterintuitive given the notably skewed ML fraction dominance of AOB described above and may indicate a degree of functional redundancy in mixed liquor AOB communities here. Interestingly, others suggest that functional redundancy in microbial communities may be important for maintaining functional stability in AS performance [41]. The lack of detectable relationships between archaeal *amoA* abundance and plant performance data also brings into question the contribution made by AOA to ammonia oxidation in our IFAS reactor; the role of AOA here is particularly unclear in the context of recent research indicating that not all AOA are obligate chemolithoautotrophs [28,29]. Adding another layer of complexity to the above interpretations is the fact that there are notable differences in organism size, cellular NH_4_^+^-N oxidation capacity and per-cell functional gene abundance between AOB and AOA (i.e. AOA are between 10 and >100-fold smaller than known AOB, have much lower per-cell NH_4_^+^-N oxidation activities and contain only one *amoA* copy as compared to 2–3 *amoA* copies per cell for AOB) [29,46]. The above issues, combined with the possibility of AOB or AOA groups that are unaccounted for by currently used PCR primer sets [24] makes tenuous any plant performance inferences drawn solely from bacterial–archaeal *amoA* gene abundance data at the present time. Robust future assessments of this nature will require assays of relative physiological activity between the two groups (e.g., via stable isotopes or mRNA transcript analyses) in parallel to comprehensive ecological data from tools such as the FGA here. 

Against the backdrop of apparently similar AOB community ecologies in both ML and media fractions, these results serve to demonstrate the dynamic nature of biological interactions between the two IFAS reactor compartments, wherein changes in the relative abundance of AOB and AOA in both phases are not necessarily reflected in community diversity and *vice versa*. Results here contrasting the FGA outputs with quantitative functional gene abundance data of qPCR highlight the fact that neither approach will encompass both aspects of community change when used in isolation. If, as speculated, community diversity is indeed an important factor in bioreactor performance [1,4], then abundance measures alone may fall short of explaining observed variations in plant performance. As such, FGA and qPCR should be viewed as complementary tools and should ideally be used in an integrated manner to gain a more holistic understanding of the ecological underpinnings of wastewater treatment process performance. 

### Practical applications of the FGA

Results presented here demonstrate the novel ammonia oxidiser FGA to be an effective and promising monitoring tool for assessing the diversity and relative abundance of AOB and AOA in nitrifying activated sludge. The potential benefits arising from the application of molecular biology tools to better understand wastewater treatment processes have been recognised for some time. In particular, diagnostics and troubleshooting for improved reliability of engineered treatment systems are seen as the first and perhaps most promising applications for these tools [1,2,4]. There is considerable current interest in microarrays in particular as the tool of choice for activated sludge monitoring; this relates to their inherent advantages over other molecular techniques, including ease of analysis, high-throughput and low cost [4,8,47]. These combined advantages make the microarray particularly suited to routine monitoring of highly dynamic environments such as wastewater bioreactors, where rapid, high-throughput analyses are required for process control purposes. Longer-term industry benefits may emerge from the integration of rapid, sensitive and potentially standardised tools such as FGAs into more routine monitoring programs and will come in the form of an improved fundamental understanding of the microbiology and microbial ecology of engineered systems and subsequent improvements in process design and operational performance. 

While the physicochemical outcomes of complex biological interactions within activated sludge systems are well characterised by mechanistic models, these tools have been constructed from a broad information base—often without a ‘microbial community’ [1,4]—and hence are designed to model a suite of generic parameters, frequently under steady-state conditions [48]. The broad spectrum ‘equilibrium’ nature of these models in particular makes them less capable of predicting plant performance under the inherently dynamic, non-steady-state at which engineered bioreactors operate [42] or under situations of notable operational instability or malfunction [1]. Prior investigations into the ecology of ammonia oxidisers in wastewater treatment systems internationally have demonstrated the overtly site-specific nature of these communities; community composition and diversity often varies widely from site-to-site and there is a growing body of evidence for tight habitat–phylogeny associations in AOA specifically. The broad spectrum, spatially-integrated nature of current approaches for designing, operating and optimising activated sludge systems do not allow for detailed insights into the nature of individual treatment systems at the community level. Such insights can only be gained through comprehensive characterisations of functional microbial ecology on a case-by-case basis and these investigations will require tools like the FGA described here. 

Ecology is widely considered to be the long-term intellectual foundation for furthering the development of bio-engineered technologies for wastewater treatment [2-4]. A comprehensive understanding of the microbial ecology of AS systems should ultimately provide a better understanding of how microbial ecology relates to process stability and performance of these systems and may ultimately allow engineers to fully exploit these technologies through informed manipulations of particular operational and/or design parameters. Demonstration projects using FGAs to successfully troubleshoot AS systems will be the next stage in documenting the immediate value of these tools to bioprocess engineers. 

## Conclusions

A novel functional gene microarray targeting ammonia oxidisers (bacterial and archaeal) was applied to probe the functional ecology of a full-scale integrated fixed-film activated sludge plant. From a practical perspective, the novel FGA tool performed well in the environmental wastewater sample matrices tested. AOA were found to be ubiquitous in all IFAS reactor samples analysed; this is in contrast to some prior studies which have failed to recover archaeal ammonia oxidisers from wastewater systems. AOB diversity was shown to be similar in the ML and media fractions of the IFAS reactor, whereas AOA community diversity was found to differ significantly between the two reactor compartments. Contrasting analysis of amoA functional gene abundance by qPCR revealed a dominance of AOB over AOA in the ML phase; however, this trend was moderated in the media fraction despite the apparent functional importance of media-attached AOB to IFAS plant nitrification performance. Overall results from this study demonstrate the capability of the FGA as a rapid, high resolution, high-throughput ecological monitoring tool for studying key microbial communities in nitrifying activated sludge and indicate its potential future value as a tool for better understanding the links between ecology and performance in advanced biological wastewater treatment systems.

## References

[1] Briones A, Raskin L (2003) Diversity and dynamics of microbial communities in engineered environments and their implications for process stability. Curr Opin Biotechnol 14: 270–276. doi:10.1016/S0958-1669(03)00065-X. PubMed: 12849779. 12849779

[2] Oerther D, Love N (2003) The value of applying molecular biology tools in environmental engineering: Academic and industry perspective in the USA. Rev Environ Sci Biotechnol 2: 1–8. doi:10.1023/B:RESB.0000022932.74077.5e.

[3] Satoh H, Yamakawa T, Kindaichi T, Ito T, Okabe S (2006) Community structures and activities of nitrifying and denitrifying bacteria in industrial wastewater-treating biofilms. Biotechnol Bioeng 94: 762–772. doi:10.1002/bit.20894. PubMed: 16477661. 16477661

[4] Seviour R, Nielsen PH, editors (2010) Microbial Ecology of Activated Sludge. London, UK: IWA Publishing ISBN: 9781843390329.

[5] Okabe S, Yoshiteru A, Hisashi S, Suwa Y (2011) Nitrification in wastewater treatment. In: Ward BB Arp DJ Klotz MG Nitrification. Washington, DC: ASM Press p. ISBN-13. p. 978-1-55581-481-6. pp. 405–433

[6] Hatzenpichler R (2012) Diversity, Physiology, and Niche Differentiation of Ammonia-Oxidizing Archaea. Appl Environ Microbiol 78: 7501–7510. doi:10.1128/AEM.01960-12. PubMed: 22923400. 22923400 PMC3485721

[7] Zhang T, Jin T, Yan Q, Shao M, Wells G et al. (2009) Occurrence of ammonia-oxidizing Archaea in activated sludges of a laboratory scale reactor and two wastewater treatment plants. J Appl Microbiol 107: 970–977. doi:10.1111/j.1365-2672.2009.04283.x. PubMed: 19486399. 19486399

[8] Roh SW, Abell GCJ, Kim KH, Nam YD, Bae JW (2010) Comparing microarrays and next-generation sequencing technologies for microbial ecology research. Trends Biotechnol 28: 291–299. doi:10.1016/j.tibtech.2010.03.001. PubMed: 20381183. 20381183

[9] Schena M, Shalon D, Davis RW, Brown PO (1995) Quantitative monitoring of gene expression patterns with a complementary DNA microarray. Science 270: 467–470. doi:10.1126/science.270.5235.467. PubMed: 7569999. 7569999

[10] Adamczyk J, Hesselsoe M, Iversen N, Horn M, Lehner A et al. (2003) The isotope array, a new tool that employs substrate-mediated labeling of rRNA for determination of microbial community structure and function. Appl Environ Microbiol 69: 6875–6887. doi:10.1128/AEM.69.11.6875-6887.2003. PubMed: 14602652. 14602652 PMC262286

[11] Kelly JJ, Siripong S, McCormack J, Janus LR, Urakawa H et al. (2005) DNA microarray detection of nitrifying bacterial 16S rRNA in wastewater treatment plant samples. Water Res 39: 3229–3238. doi:10.1016/j.watres.2005.05.044. PubMed: 16009395. 16009395

[12] Siripong S, Kelly JJ, Stahl DA, Rittmann BE (2006) Impact of prehybridization PCR amplification on microarray detection of nitrifying bacteria in wastewater treatment plant samples. Environ Microbiol 8: 1564–1574. doi:10.1111/j.1462-2920.2006.01047.x. PubMed: 16913917. 16913917

[13] Abell GCJ, Robert SS, Frampton DMF, Volkman JK, Rizwi F et al. (2012) High-Throughput Analysis of Ammonia Oxidiser Community Composition via a Novel, *amoA*-Based Functional Gene Array. PLOS ONE 7: e51542. doi:10.1371/journal.pone.0051542. PubMed: 23284709. 23284709 PMC3526613

[14] van den Akker B, Beard H, Kaeding U, Giglio S, Short MD (2010) Exploring the relationship between viscous bulking and ammonia-oxidiser abundance in activated sludge: A comparison of conventional and IFAS systems. Water Res 44: 2919–2929. doi:10.1016/j.watres.2010.02.016. PubMed: 20202664. 20202664

[15] Abell GC, Revill AT, Smith C, Bissett AP, Volkman JK et al. (2010) Archaeal ammonia oxidizers and nirS-type denitrifiers dominate sediment nitrifying and denitrifying populations in a subtropical macrotidal estuary. ISME J 4: 286–300. doi:10.1038/ismej.2009.105. PubMed: 19798039. 19798039

[16] Stralis-Pavese N, Abell GCJ, Sessitsch A, Bodrossy L (2011) Analysis of methanotroph community composition using a *pmoA*-based microbial diagnostic microarray. Nat Protoc 6: 609–624. doi:10.1038/nnano.2011.174. PubMed: 21527919. 21527919

[17] Abell GCJ, Banks J, Ross DJ, Keane JP, Robert SS et al. (2011) Effects of estuarine sediment hypoxia on nitrogen fluxes and ammonia oxidizer gene transcription. FEMS Microbiol Ecol 75: 111–122. doi:10.1111/j.1574-6941.2010.00988.x. PubMed: 21083579. 21083579

[18] Clarke KR, Ainsworth M (1993) A method of linking multivariate community structure to environmental variables. Mar Ecol Prog S 92: 205–219. doi:10.3354/meps092205.

[19] R-Development-Core-Team (2010) R: A language and environment for statistical computing. Austria: Vienna.

[20] Biller SJ, Mosier AC, Wells GF, Francis CA (2012) Global biodiversity of aquatic ammonia-oxidizing archaea is partitioned by habitat. Frontiers in Microbiology. p. 3: 252 10.3389/fmicb.2012.00252 PMC3399221 22826704

[21] Park HD, Wells GF, Bae H, Criddle CS, Francis CA (2006) Occurrence of ammonia-oxidizing archaea in wastewater treatment plant bioreactors. Appl Environ Microbiol 72: 5643–5647. doi:10.1128/AEM.00402-06. PubMed: 16885322. 16885322 PMC1538709

[22] Sauder LA, Peterse F, Schouten S, Neufeld JD (2012) Low-ammonia niche of ammonia-oxidizing archaea in rotating biological contactors of a municipal wastewater treatment plant. Environ Microbiol 14: 2589–2600. doi:10.1111/j.1462-2920.2012.02786.x. PubMed: 22639927. 22639927 PMC3466407

[23] Fernàndez-Guerra A, Casamayor EO (2012) Habitat-Associated Phylogenetic Community Patterns of Microbial Ammonia Oxidizers. PLOS ONE 7: e47330. doi:10.1371/journal.pone.0047330. PubMed: 23056629. 23056629 PMC3467245

[24] Erguder TH, Boon N, Wittebolle L, Marzorati M, Verstraete W (2009) Environmental factors shaping the ecological niches of ammonia-oxidizing archaea. FEMS Microbiol Rev 33: 855–869. doi:10.1111/j.1574-6976.2009.00179.x. PubMed: 19453522. 19453522

[25] Martens-Habbena W, Berube PM, Urakawa H, de la Torre JR, Stahl DA (2009) Ammonia oxidation kinetics determine niche separation of nitrifying Archaea and Bacteria. Nature 461: 976–979. doi:10.1038/nature08465. PubMed: 19794413. 19794413

[26] Bai Y, Sun Q, Wen D, Tang X (2012) Abundance of ammonia-oxidizing bacteria and archaea in industrial and domestic wastewater treatment systems. FEMS Microbiol Ecol 80: 323–330. doi:10.1111/j.1574-6941.2012.01296.x. PubMed: 22611552. 22611552

[27] Kayee P, Sonthiphand P, Rongsayamanont C, Limpiyakorn T (2011) Archaeal *amoA* Genes Outnumber Bacterial *amoA* Genes in Municipal Wastewater Treatment Plants in Bangkok. Microb Ecol 62: 1–13. doi:10.3354/ame01451. PubMed: 21331609. 21706196

[28] Mußmann M, Brito I, Pitcher A, Sinninghe Damsté JS, Hatzenpichler R et al. (2011) Thaumarchaeotes abundant in refinery nitrifying sludges express *amoA* but are not obligate autotrophic ammonia oxidizers. Proc Natl Acad Sci U_S_A 108: 16771–16776. doi:10.1073/pnas.1106427108. PubMed: 21930919. 21930919 PMC3189051

[29] Nicol GW, Leininger S, Schleper C (2011) Distribution and activity of ammonia-oxidizing Archaea in natural environments. In: Ward BB Arp DJ Klotz MG Nitrification. Washington, DC: ASM Press p. ISBN-13. p. 978-1-55581-481-6. pp. 157–178

[30] Blainey PC, Mosier AC, Potanina A, Francis CA, Quake SR (2011) Genome of a Low-Salinity Ammonia-Oxidizing Archaeon Determined by Single-Cell and Metagenomic Analysis. PLOS ONE 6: e16626. doi:10.1371/journal.pone.0016626. PubMed: 21364937. 21364937 PMC3043068

[31] Kim HS, Schuler AJ, Gunsch CK, Pei R, Gellner J et al. (2011) Comparison of Conventional and Integrated Fixed-Film Activated Sludge Systems: Attached- and Suspended-Growth Functions and Quantitative Polymerase Chain Reaction Measurements. Water Environ Res 83: 627–635. doi:10.2175/106143010X12851009156448. PubMed: 21790081. 21790081

[32] Li C, Li XL, Ji M, Liu J (2012) Performance and microbial characteristics of integrated fixed-film activated sludge system treating industrial wastewater. Water Sci Technol 66: 2785–2792. doi:10.2166/wst.2012.421. PubMed: 23109599. 23109599

[33] Mahendran B, Lishman L, Liss SN (2012) Structural, physicochemical and microbial properties of flocs and biofilms in integrated fixed-film activated sludge (IFFAS) systems. Water Res 46: 5085–5101. doi:10.1016/j.watres.2012.05.058. PubMed: 22832219. 22832219

[34] Onnis-Hayden A, Majed N, Schramm A, Gu AZ (2011) Process optimization by decoupled control of key microbial populations: Distribution of activity and abundance of polyphosphate-accumulating organisms and nitrifying populations in a full-scale IFAS-EBPR plant. Water Res 45: 3845–3854. doi:10.1016/j.watres.2011.04.039. PubMed: 21641011. 21641011

[35] Xia S, Li J, Wang R (2008) Nitrogen removal performance and microbial community structure dynamics response to carbon nitrogen ratio in a compact suspended carrier biofilm reactor. Ecol Eng 32: 256–262. doi:10.1016/j.ecoleng.2007.11.013.

[36] Jin T, Zhang T, Yan Q (2010) Characterization and quantification of ammonia-oxidizing archaea (AOA) and bacteria (AOB) in a nitrogen-removing reactor using T-RFLP and qPCR. Appl Microbiol Biotechnol 87: 1167–1176. doi:10.1007/s00253-010-2595-2. PubMed: 20405121. 20405121 PMC2886134

[37] Limpiyakorn T, Sonthiphand P, Rongsayamanont C, Polprasert C (2011) Abundance of *amoA* genes of ammonia-oxidizing archaea and bacteria in activated sludge of full-scale wastewater treatment plants. Bioresour Technol 102: 3694–3701. doi:10.1016/j.biortech.2010.11.085. PubMed: 21185720. 21185720

[38] French E, Kozlowski JA, Mukherjee M, Bullerjahn G, Bollmann A (2012) Ecophysiological Characterization of Ammonia-Oxidizing Archaea and Bacteria from Freshwater. Appl Environ Microbiol 78: 5773–5780. doi:10.1128/AEM.00432-12. PubMed: 22685142. 22685142 PMC3406153

[39] Gapes DJ, Keller J (2009) Impact of oxygen mass transfer on nitrification reactions in suspended carrier reactor biofilms. Proc Biochem 44: 43–53. doi:10.1016/j.procbio.2008.09.004.

[40] Limpiyakorn T, Fürhacker M, Haberl R, Chodanon T, Srithep P et al. (2013) *amoA*-encoding archaea in wastewater treatment plants: a review. Appl Microbiol Biotechnol 97: 1425–1439. doi:10.1007/s00253-012-4650-7. PubMed: 23306641. 23306641

[41] Valentín-Vargas A, Toro-Labrador G, Massol-Deyá AA (2012) Bacterial Community Dynamics in Full-Scale Activated Sludge Bioreactors: Operational and Ecological Factors Driving Community Assembly and Performance. PLOS ONE 7: e42524. doi:10.1371/journal.pone.0042524. PubMed: 22880016. 22880016 PMC3411768

[42] Wells GF, Park HD, Eggleston B, Francis CA, Criddle CS (2011) Fine-scale bacterial community dynamics and the taxa–time relationship within a full-scale activated sludge bioreactor. Water Res 45: 5476–5488. doi:10.1016/j.watres.2011.08.006. PubMed: 21875739. 21875739

[43] Ofiteru ID, Lunn M, Curtis TP, Wells GF, Criddle CS et al. (2010) Combined niche and neutral effects in a microbial wastewater treatment community. Proc Natl Acad Sci U_S_A 107: 15345–15350. doi:10.1073/pnas.1000604107. PubMed: 20705897. 20705897 PMC2932620

[44] Winkler MK, Kleerebezem R, Bruin LMM, Verheijen PJT, Abbas B et al. (2013) Microbial diversity differences within aerobic granular sludge and activated sludge flocs. Appl Microbiol Biotechnol 97: 7447–7458. doi:10.1007/s00253-012-4472-7. PubMed: 23064482. 23064482

[45] Regmi P, Thomas W, Schafran G, Bott C, Rutherford B et al. (2011) Nitrogen removal assessment through nitrification rates and media biofilm accumulation in an IFAS process demonstration study. Water Res 45: 6699–6708. doi:10.1016/j.watres.2011.10.009. PubMed: 22040713. 22040713

[46] Santoro AE, Casciotti KL, Francis CA (2010) Activity, abundance and diversity of nitrifying archaea and bacteria in the central California Current. Environ Microbiol 12: 1989–2006. doi:10.1111/j.1462-2920.2010.02205.x. PubMed: 20345944. 20345944

[47] DeSantis TZ, Brodie EL, Moberg JP, Zubieta IX, Piceno YM et al. (2007) High-density universal 16S rRNA microarray analysis reveals broader diversity than typical clone library when sampling the environment. Microb Ecol 53: 371–383. doi:10.1007/s00248-006-9134-9. PubMed: 17334858. 17334858

[48] Melcer H, Dold PL, Jones RM, Bye CM, Takacs I et al. (2003) Methods for Wastewater Characterization in Activated Sludge Modelling. Water Environment Research Foundarion (WERF), Report 99 -WWF-3. ISBN: 1-893664-71-6

